# Postnatal Environmental Tobacco Smoke Exposure Related to Behavioral Problems in Children

**DOI:** 10.1371/journal.pone.0133604

**Published:** 2015-08-05

**Authors:** Julie Chastang, Nour Baïz, Jean Sébastien Cadwalladder, Sarah Robert, John Dywer, Denis André Charpin, Denis Caillaud, Frédéric de Blay, Chantal Raherison, François Lavaud, Isabella Annesi-Maesano

**Affiliations:** 1 EPAR, INSERM UMR-S 1136, Paris, France; 2 Department of General Practice, UPMC Université Paris 6, School of Medicine, Paris, France; 3 EPAR, Université Pierre et Marie Curie, UMR-S 1136, Paris, France; 4 Service de Pneumologie, CHU Marseille, Marseille, France; 5 CHU Clermont-Ferrand, Clermont-Ferrand, France; 6 CHU Strasbourg, Strasbourg, France; 7 CHU Bordeaux, Bordeaux, France; 8 CHU Reims, Reims, France; Chiba University Center for Forensic Mental Health, JAPAN

## Abstract

**Objective:**

The purpose of this study was to examine the association between pre and post environmental tobacco smoke (ETS) exposure and behavioral problems in schoolchildren.

**Methods:**

In the cross-sectional 6 cities Study conducted in France, 5221 primary school children were investigated. Pre- and postnatal exposure to secondhand tobacco smoke at home was assessed using a parent questionnaire. Child’s behavioral outcomes (emotional symptoms and conduct problems) were evaluated by the Strengths and Difficulties Questionnaire (SDQ) completed by the parents.

**Results:**

ETS exposure during the postnatal period and during both pre- and postnatal periods was associated with behavioral problems in children. Abnormal emotional symptoms (internalizing problems) were related to ETS exposure in children who were exposed during the pre- and postnatal periods with an OR of 1.72 (95% Confidence Interval (CI)= 1.36-2.17), whereas the OR was estimated to be 1.38 (95% CI= 1.12-1.69) in the case of postnatal exposure only. Abnormal conduct problems (externalizing problems) were related to ETS exposure in children who were exposed during the pre- and postnatal periods with an OR of 1.94 (95% CI= 1.51-2.50), whereas the OR was estimated to be 1.47 (95% CI=1.17-1.84) in the case of postnatal exposure only. Effect estimates were adjusted for gender, study center, ethnic origin, child age, low parental education, current physician diagnosed asthma, siblings, preterm birth and single parenthood.

**Conclusion:**

Postnatal ETS exposure, alone or in association with prenatal exposure, increases the risk of behavioral problems in school-age children.

## Introduction

The consequences of childhood environmental tobacco smoke (ETS) exposure have often been described [1, 2] and include many physical symptoms or diseases such as asthma or sudden infant death syndrome. However, much less is known about the potential role of ETS exposure in the development of behavioral problems in children. Association between behavioral problems and ETS exposure during fetal development has been suggested in several studies [3–5]. Recently, a dose-response relationship was reported between postnatal ETS exposure at home and hyperactivity/inattention as well as conduct problems in preschool children [6]. Furthermore, in a prospective birth cohort study, Tiesler et al. investigates the impact of passive smoking on behavioral problems. In this study, they found that not only maternal smoking during pregnancy but also paternal smoking at home is associated with hyperactivity/inattention problems in children [7].

Few studies have investigated the relationship between postnatal ETS and emotional symptoms or conduct problems. The purpose of this study was to investigate, in a large population-based sample of children and using internationally referenced instruments, the relationships between behavioral problems (emotional symptoms and conduct problems) and exposure to pre- and mostly postnatal ETS exposure.

## Materials and Methods

### Participants

9615 children were recruited in primary school (CM1 and CM2 in France) in the frame of the French 6 Cities Study (6C Study) according to a protocol described in a previous study [8]. The sample was taken from all pupils in the 401 relevant classes from 108 schools randomly selected in the six French communities (Bordeaux, Clermont-Ferrand, Creteil, Marseille, Strasbourg and Reims), which were chosen for the contrast in their air quality.

7781 questionnaires have been collected. A total of 5221 children (54.3%), for whom complete data on ETS exposure and at least one of the two outcome variables (emotional symptoms or conduct problems) were available, have been included in the present study.

### Behavioral problems

The Strengths and Difficulties Questionnaire (SDQ) is a validated questionnaire used to assess mental and behavioral strengths and difficulties in 3–16 years old children, which has been endorsed in France [9]. All the questionnaires were completed by the parents of the children. Emotional symptoms and conduct problems were measured through the SDQ on childrens’ behavior in the past 6 months. In each scale, five items were scored, using a three-point Likert scale: 0 for « not true », 1 for « somewhat true » or 2 for « very true » and summed up into score ranging from 0 to 10. According to the normative banding method for parent-reported SDQ scores in France [9], the scores were categorized to « normal », « borderline » or « abnormal » using the following cut-off points: 0–3, 4 and 5–10 respectively for emotional problems and 0–2, 3, 4–10 for conduct problems.

### Exposure to environmental tobacco smoke (ETS)

Active smoking behavior of the mother, the father and any other household members at home during pregnancy, at 1 year of age and at the moment of the study was reported in the questionnaire. Children were defined as « never » being exposed to ETS when the mother reported no smoking during pregnancy, and when no smoking at home (mother, father and other members) was reported at 1 year of age and at the moment of the study.

Children were classified as being only prenatally exposed to ETS when the mother reported smoking during pregnancy but no smoking at home was reported at 1 year of age and at the moment of the study. Children were classified as being only postnatally exposed to ETS when smoking at home at 1 year of age or at the moment of the study was reported, but when the mother did not smoke during pregnancy. Pre- and postnatal ETS exposure was defined for children whose mothers had smoked during pregnancy and whose family had reported smoking at home at 1 year of age or at the moment of the study.

### Statistical analysis

The characteristics of our study population (N = 5221) were compared to the sample of children without complete data (N = 2560), by using Chi-square tests. We also compared these characteristics in children according to their emotional symptoms and conduct problems, using Kruskal-Wallis tests.

In the unadjusted models, a total of 5077 children were included in the analyses of emotional symptoms and of 5126 children in the analyses of conduct problems.

We used a multinomial logistic regression model to analyze the association between behavioral problems and ETS exposure [10]. The dependent variables (emotional symptoms and conduct problems) were classified in three categories (normal, borderline and abnormal). Results are presented as odds ratios (OR) and 95% confidence intervals (95% CI). Covariate selection was based on the statistical significance of comparison tests between our study population and the rest of the population, and based on the known relationships to behavioral problems and/or ETS exposure. Parental education was defined as high if both parents attained tertiary level and low otherwise (primary and/or secondary). Children were considered to have a recent asthma diagnosis if they had been diagnosed by a doctor with asthma in the last 12 months. The variable “siblings” was classified into “presence of one or more siblings” and “no sibling”. Preterm birth was defined as a live birth before 37 completed weeks of gestation.

The final models were adjusted for gender, study center, ethnic origin, child age, low parental education, current physician diagnosed asthma, siblings, preterm birth and single parenthood.

In addition, interactions between ETS exposure and the covariates have been tested.

Dataset used in this work is given in S1 Dataset. All statistical analyses were performed using the statistical software SAS version 9.3 (SAS Institute Inc., Cary, NC, USA).

### Ethics

This study was approved by the French national Ethics Board (C.C.P.P.R.B., approval number: 01/07, Marseille AP, France). Parents signed a formal consent after having been informed about the study.

## Results

### Characteristics of the study population

Children were included in the analyses if their parents had participated and answered questions about smoking behavior and completed the SDQ (5221 children).

These 5221 children differ in many characteristics from children with missing data (N = 2560), including children age, parents’ educational level, asthma, siblings, single parenthood, preterm birth, origin and city (Table 1). They were aged 10.8 +/- 0.8 in mean. 2619 children of our study population (50%) were girls (Table 1).

**Table 1:** Comparison of basic characteristics of our study population with complete data (N = 5221) and the rest of the population without complete data (N = 2560).

	Study sample with complete data n/N (%)	Sample without complete data n/N (%)	Chi-square test *P-value*
**Gender**			
Male	2602/5221 (49.8)	1270/2559 (49.6)	0.86
Fermale	2619/5221 (50.1)	1289/2559 (50.4)	
**Study center**			
Creteil	912/5221 (17.5)	738/2560 (28.8)	<0.0001
Reims	659/5221 (12.6)	396/2560 (15.5)	
Marseille	816/5221 (15.6)	411/2560 (16.1)	
Strasbourg	904/5221 (17.3)	317/2560 (12.4)	
Clermont-Ferrand	969/5221 (18.6)	316/2560 (12.3)	
Bordeaux	961/5221 (18.4)	382/2560 (14.3)	
**Passive smoking**			
ETS only prenatal	43/5221 (0.8)	0/97 (0.0)	
ETS only postnatal	1973/5221 (37.8)	33/97 (34.0)	
ETS pre and postnatal	1132/5221 (21.7)	18/97 (18.6)	
None	2073/5221 (39.7)	46/97 (47.4)	0.39
**Child age (in years)**			
8-9-10	2011/5221 (38.5)	1267/2560 (49.5)	<0.0001
11	2389/5221 (45.8)	833/2560 (32.5)	
12-13-14	821/5221 (15.7)	460/2560 (18.0)	
**Recent doctor-diagnosed asthma**			
Yes	253/5079 (5.0)	65/1838 (3.5)	0.0112
No	4826/5079 (95.0)	1773/1838 (96.5)	
**Parental attainment (the highest educational attainment of both fathers and mothers)**			
High (tertiary)	2581/4892 (52.8)	543/1622 (33.5)	<0.0001
Low (primary or secondary)	2311/4892 (47.2)	1079/1622 (66.5)	
**Ethnic origin**			
French overseas and foreign countries	781/5080 (15.4)	751/1831 (41.0)	<0.0001
Metropolitan France	4299/5080 (84.6)	1080/1831 (59.0)	
**Siblings**			
Yes	3994/5221 (76.5)	1244/2560 (48.6)	<0.0001
No	1227/5221 (23.5)	1316/2560 (51.4)	
**Preterm birth**			
Yes	1317/5042 (26.1)	306/1641 (18.7)	<0.0001
No	3725/5042 (73.9)	1335/1641 (81.4)	
**Single parent**			
Yes	1075/5200 (20.7)	591/1915 (30.9)	<0.0001
No	4125/5200 (79.3)	1324/1915 (69.1)	

Table 2 presents the characteristics of the sample of 5221 children of our study according to emotional symptoms and conduct problems.

**Table 2:** Characteristics of the study population according to emotional symptoms (internalizing problems) and conduct problems (externalizing problems).

	Emotional problems (N = 5077)n (%)			Conduct problems (N = 5126)n (%)		
	Normal3785 (75.5)	Borderline541 (10.7)	Abnormal751 (14.8)	Normal3761 (73.4)	Borderline687 (13.4)	Abnormal678 (13.2)
Gender (*/***) †						
Male	1918(75.8)	275(10.9)	336(13.3)	1710(66.8)	413(16.1)	436(17.0)
Fermale	1867(73.3)	266(10.4)	415(16.3)	2051(79.9)	274(10.7)	242(9.4)
Study Center (ns/**)						
Creteil	661(74.4)	95(10.7)	133(15.0)	678(75.6)	105(11.7)	114(12.7)
Reims	460(71.3)	75(11.6)	110(17.1)	437(67.7)	95(14.7)	114(17.7)
Marseille	579(76.7)	81(10.5)	112(14.5)	566(71.6)	107(13.5)	118(14.9)
Strasbourg	679(76.7)	89(10.1)	117(13.2)	652(73.3)	125(14.0)	113(12.7)
Clermont-Ferrand	701(74.2)	101(10.7)	143(15.1)	720(75.2)	117(12.2)	120(12.5)
Bordeaux	705(74.9)	100(10.6)	136(14.5)	708(74.9)	138(14.6)	99(10.5)
Passive smoking (***/***)						
ETS only prenatal	28(66.7)	5(11.9)	9(21.4)	34(81.0)	6(14.3)	2(4.8)
ETS only postnatal	1387(72.7)	218(11.4)	304(15.9)	1387(71.6)	271(14.0)	278(14.4)
ETS pre and postnatal	765(69.6)	130(11.8)	205(18.6)	715(64.8)	189(17.1)	200(18.1)
None	1605(79.2)	188(9.3)	233(11.5)	1625(79.5)	221(10.8)	198(9.7)
Child age (in years) (*/***)						
8-9-10	1489(76.0)	191(9.8)	279(14.2)	1482(75.1)	256(13.0)	235(11.9)
11	1744(74.8)	256(11.0)	333(14.3)	1747(74.2)	322(13.7)	286(12.1)
12-13-14	552(70.3)	94(12.0)	139(17.7)	532(66.7)	109(13.7)	157(19.7)
Recent doctor-diagnosed asthma (***/ns)						
Yes	154(61.9)	37(14.9)	58(23.3)	170(69.7)	31(12.7)	43(17.6)
No	3543(75.4)	485(10.3)	670(14.3)	3499(73.8)	634(13.4)	610(12.9)
Parental attainment (the highest educational attainment of both fathers and mothers) (***/***)						
High (tertiary)	1953(77.2)	239(9.4)	339(13.4)	1560(69.0)	328(14.5)	374(16.5)
Low (primary or secondary)	1604(72.0)	262(11.8)	362(16.3)	1997(78.5)	304(12.0)	242(9.5)
**Ethnic origin (ns/ns)**						
French overseas and foreign countries	571(76.1)	71(9.5)	108(14.4)	544(70.8)	108(14.1)	116(15.1)
Metropolitan France	3116(74.2)	460(11.0)	623(14.8)	3134(74.2)	557(13.2)	532(12.6)
**Siblings (ns/ns)**						
Yes	2905(74.7)	427(11.0)	556(14.3)	2900(73.8)	525(13.4)	506(12.9)
No	880(74.0)	114(9.6)	195(16.4)	861(72.1)	162(13.6)	172(14.4)
**Preterm birth (ns/ns)**						
Yes	933(72.6)	145(11.3)	208(16.2)	942(72.7)	163(12.6)	191(14.7)
No	2728(75.3)	379(10.5)	515(14.2)	2703(73.9)	493(13.5)	460(12.6)
Single Parent (***/***)						
Yes	731(70.0)	121(11.6)	192(18.4)	691(65.6)	173(16.4)	190(18.0)
No	3034(75.6)	419(10.4)	559(13.9)	3056(75.4)	512(12.6)	484(11.9)

^†^ (p-value of Chi-square test for emotional problems / p-value of Chi-square test for conduct problems).

Chi-square test:

* *P*<0.05,

** *P*<0.01,

*** *P*<0.001,

ns: non-significant.

### ETS exposure and behavioral problems outcomes

Around 1% of the children were exposed during the prenatal period only, whereas 38% were exposed during the postnatal period only. 21% of the children were exposed to tobacco smoke in the both pre- and the postnatal period (Table 1).

Emotional symptoms were classified as “borderline” in 10.7% of the children and “abnormal” in 14.8%, whereas conduct problems were “borderline” in 13.4% of the children and “abnormal” in 13.2% (Table 2).

### Associations between ETS exposure in children and behavioral problems

Unadjusted and sex-adjusted logistic regression models showed that compared to children who were never exposed to ETS, children who exposed to ETS had higher risk of behavioral problems at 10 years of age. More specifically, ETS exposure (prenatally only, postnatally only and both prenatally and postnatally) was associated with a higher risk of borderline and abnormal emotional symptoms, although not significantly for borderline emotional symptoms in children exposed to ETS only prenatally (Table 3). In addition, ETS exposure during the postnatal period and the whole period (pre and postnatal) was significantly associated with a higher risk of “borderline” and “abnormal” conduct problems and effects of ETS exposure on conduct problems were stronger when children were exposed during pregnancy and the postnatal period (Table 3). ETS exposure only prenatally was not significantly associated with conduct problems (Table 3). Indeed, it may be possible that the very low sample of children exposed to prenatal ETS (N = 34) did not allow us to draw any conclusion concerning prenatal exposure. After adjustment for gender, study center, ethnic origin, child age, low parental education, current physician diagnosed asthma, siblings, preterm birth and single parenthood, results persisted for “abnormal” emotional symptoms and for both “borderline” and “abnormal” conduct problems (Table 3) in children who were exposed to ETS postnatally only and pre- and postnatally. “Abnormal” emotional symptoms were related to ETS exposure in children who were exposed during the postnatal period, with an OR of 1.38 (95% CI = 1.12–1.69) and during both the pre- and postnatal periods with an OR of 1.72 (95% CI = 1.36–2.17). “Borderline” and “abnormal” conduct problems were related to ETS exposure in children who were exposed during the postnatal period with an OR of 1.32 (95% CI = 1.06–1.63) and 1.47 (95% CI = 1.17–1.84) respectively and during both the pre- and postnatal periods with an OR of 1.73 (95% CI = 1.36–2.21) and 1.94 (95% CI = 1.51–2.50) respectively.

**Table 3:** Associations between emotional symptoms (internalizing problems), conduct problems (externalizing problems) and ETS exposure: results from multinomial logistic regression analysis.

ETS	Never(ref)	Only prenatal	Only postnatal	Pre and postnatal
	OR	OR (95% CI)	OR (95% CI)	OR (95% CI)
**Emotional Symptoms**				
**Unadjusted**	N = 2026	N = 42	N = 1909	N = 1100
Borderline	1.00	1.53 (0.52–4.00)	**1.34 (1.09–1.65)**	**1.45 (1.14–1.84)**
Abnormal	1.00	**2.22 (1.03–4.76)**	**1.51 (1.25–1.82)**	**1.85 (1.50–2.27)**
**Sex-adjusted**	N = 2026	N = 42	N = 1909	N = 1100
Borderline	1.00	1.52 (0.58–4.00)	**1.34 (1.09–1.65)**	**1.45 (1.14–1.84)**
Abnormal	1.00	**2.25 (1.05–4.83)**	**1.51 (1.25–1.82**)	**1.86 (1.51–2.29)**
Multi-adjusted*	N = 1775	N = 35	N = 1660	N = 953
Borderline	1.00	1.28 (0.44–3.75)	1.24 (0.99–1.56)	1.24 (0.94–1.62)
Abnormal	1.00	1.61 (0.64–4.00)	**1.38 (1.12–1.69)**	**1.72 (1.36–2.17)**
**Conduct problems**				
**Unadjusted**	N = 2044	N = 42	N = 1936	N = 1104
Borderline	1.00	1.30 (0.54–3.13)	**1.44 (1.19–1.74)**	**1.94 (1.57–2.41)**
Abnormal	1.00	0.48 (0.12–2.03)	**1.65 (1.35–2.00)**	**2.30 (1.85–2.85)**
**Sex-adjusted**	N = 2044	N = 42	N = 1936	N = 1104
Borderline	1.00	1.27 (0.53–3.08)	**1.45 (1.20–1.76)**	**1.94 (1.57–2.40)**
Abnormal	1.00	0.47 (0.11–1.99)	**1.67 (1.37–2.04)**	**2.30 (1.85–2.85)**
Multi-adjusted*	N = 1771	N = 34	N = 1673	N = 953
Borderline	1.00	1.38 (0.52–3.65)	**1.32 (1.06–1.63)**	**1.73 (1.36–2.21)**
Abnormal	1.00	0.32 (0.04–2.37)	**1.47 (1.17–1.84)**	**1.94 (1.51–2.50)**

*Adjusted for gender, study center, ethnic origin, child age, low parental education, current doctor diagnosed asthma, siblings, preterm birth, and single parent. Results in bold are statistically significant (p-value < 0.05).

When testing interactions between ETS exposure and the covariates, only the one with parental education was significant (*P* = 0.03, data not shown). We found that the relationship between emotional symptoms and ETS exposure was weaker in parents with a low educational level.

## Discussion

In our population-based sample of children, both emotional and conduct behavioral problems were related to tobacco exposure in early life and later. This association could not be explained by confounders such as gender, study center, ethnic origin, child age, low parental education, current physician diagnosed asthma, siblings, preterm birth or having a single parent. This association was stronger among children who were exposed during the pre- and postnatal periods than among children who were exposed during the postnatal period only.

### Interpretation of the results

ETS exposure appears to be associated with an increased risk of behavioral problems (emotional and conduct problems) among schoolchildren. Both pre- and postnatal exposure but also postnatal exposure alone seem to be linked with conduct problems or emotional symptoms in our study. The effect of pre- and/or postnatal ETS exposure at home and behavioral outcomes have also been assessed by Rückinger *et al*. in the German GINIplus study population [11]. In this study, the authors found significant associations between ETS exposure during the pre- and post-natal period and emotional symptoms (OR: 1.4 (95%CI: 1.0–1.9) and conduct problems (OR: 1.8 (95% CI: 1.3–2.3)). In addition, high ETS exposure during prenatal period was significantly associated with emotional symptoms.” However, no significant association was found between postnatal ETS exposure and emotional symptoms or conduct problems.

The relationship between postnatal ETS exposure and behavioral problems taking maternal smoking during pregnancy into account has been studied in a large population of preschool children in Bavaria. After adjustment for socioeconomic factors, low birth weight and maternal smoking before and during pregnancy, secondhand tobacco smoke was associated with hyperactivity/inattention as well as conduct problems [6]. In another study, 5342 mothers provided data on child behavior (emotional, conduct and social, attentional thought) when children were 5 years old [12]. The presence of a separate association between concurrent maternal smoking and externalizing child’s behavior suggested an effect of ETS postnatal exposure on child behavior problems. A fourth study examined the association of ETS exposure using serum cotinine levels [13]. In the analysis adjusted for child’s age, maternal age at child birth, child’s sex, child’s race, prenatal tobacco smoke exposure, poverty-to-income-ratio, and blood lead level, postnatal tobacco exposure was associated with an increased risk of conduct disorder (criteria of the *Diagnostic and Statistical Manual of Mental Disorders*, 4^th^ edition).

Hence our results support previous research, which had demonstrated that ETS exposure was related to increased rates of behavior problems in children. However, our findings extend previous research. The uniqueness of our study consisted in showing that in a large population-based sample of children, postnatal ETS exposure was linked with both conduct problems and emotional symptoms in the SDQ.

Only another study found similar results but it used a different rating scale and the sample was smaller than ours (N = 230) [14]. This study used data from a community based, longitudinal investigation examining the relation between childhood’s exposure to ETS and later emotional symptoms and conduct problems. Exposure to parental smoking increased the risk for their children to develop emotional symptoms and conduct problems. This relationship could not be explained by a number of psychosocial risk factors like demographic variables (parental gender, mother’s age at child’s birth, child gender, education level), parental intra-personal attributes (depression or unconventionality), and parental child-rearing practices (affection toward child). Child behavior problems were measured using items from the Child Behavior Checklist (CBCL) of Achenbach. Many studies suggest that the SDQ is significantly better than the CBCL at detecting inattention and hyperactivity, and at least as good at detecting emotional and conduct problems [15, 16].

### Potential biological mechanisms

There is a possible biological mechanism to explain the negative effect of ETS exposure on behavioral outcomes in children [17]. In animals, a mode of action of nicotine exposure during development has been shown [18]. During pregnancy, tobacco smoke exposure could have neurotoxic effects through the direct interaction of nicotine with the developing brain. Nicotine is able to stimulate the nicotinic acetylcholine receptors. The consequences are brain cell death and structural alteration in the brain [18]. Slötkin et al. extended the results in animals to the postnatal period [19]. In this study, the authors exposed rhesus monkey to ETS postnatally. Then, they examined cerebrocortical regions and the midbrain for cell damage markers and lipid peroxidation. When ETS exposure was restricted to the postnatal period, the membrane/total protein ratio, a biomarker of neurite formation, indicated potential damage to neuronal projections, accompanied by reactive sprouting.

### Strengths and limitations

Our findings have some limitations. The data of parental smoking is based on parental reports. Furthermore, prenatal exposure relates only to active smoking of the mother. This could lead to an underestimation of ETS exposure. However, parental self-report of children’s exposure to ETS has been shown to be well correlated with serum cotinine levels of the child [20]. Another limitation of our study is its cross-sectional design having collected the information on ETS retrospectively. A longitudinal study would have allowed examining the relation between children’s exposure to tobacco smoke and later emotional symptoms and conduct problems better. Our results on the data « only prenatal exposure » cannot be used because the number of children included in this category was insufficient. The association between ETS exposure and behavioral problems is difficult to prove because residual confounding may explain the association. For example, we did not take into account parental depression in our analysis. However, with regard to emotional symptoms in children, ETS and parental depression are correlated [21]. Although there are few studies looking at continuity across generations, parental behavior problems (such as parental deviance and crime) could be predictors of antisocial behavior. [22] As we have not collected the data on parental mental health, we were not able to adjust for these factors in our analysis. Although we did adjust for parental educational level, residual confounding cannot be entirely ruled out.

The 5221 children of our sample differ in many characteristics from children with missing data: educational level, asthma, siblings, being a single parent, preterm birth, origin and city. However, multivariate models have been adjusted on these variables.

Strengths of our study is that our data is based on a large sample drawn from the general population and collected from 6 cities, which may give an overall view of the impact of ETS on emotional and conduct problems in children, but results need to be interpreted and applied to the general population cautiously though. In addition, we used a validated questionnaire that allowed us to differentiate emotional and conduct problems.

## Conclusion

We observed that ETS postnatal exposure, alone or in association with prenatal exposure, increased the risk of emotional and conduct problems in children. Although additional investigation is required, these findings provide further support for anti-smoking programs within families.

## Supporting Information

S1 Dataset Minimal dataset used in this work. (XLSX)
