# Correction: Postnatal Environmental Tobacco Smoke Exposure Related to Behavioral Problems in Children

**DOI:** 10.1371/journal.pone.0138164

**Published:** 2015-09-09

**Authors:** Julie Chastang, Nour Baïz, Jean Sébastien Cadwallader, Sarah Robert, John L Dywer, Denis André Charpin, Denis Caillaud, Frédéric de Blay, Chantal Raherison, François Lavaud, Isabella Annesi-Maesano

The third and fifth authors’ names are incorrectly spelled. The correct names are: Jean Sébastien Cadwallader and John L Dywer. The correct citation is: Chastang J, Baïz N, Cadwallader JS, Robert S, Dywer JL, Charpin DA, et al. (2015) Postnatal Environmental Tobacco Smoke Exposure Related to Behavioral Problems in Children. PLoS ONE 10(8): e0133604. doi: 10.1371/journal.pone.0133604
